# Transcranial direct current stimulation: a remediation tool for the treatment of childhood congenital dyslexia?

**DOI:** 10.3389/fnhum.2013.00139

**Published:** 2013-04-22

**Authors:** Carmelo M. Vicario, Michael A. Nitsche

**Affiliations:** ^1^School of Psychology, The University of QueenslandSt. Lucia, QLD, Australia; ^2^Department of Clinical Neurophysiology, University of GöttingenGöttingen, Germany

Developmental dyslexia (DD) is a neuropsychological condition that is characterized by the persistent difficulty in learning to read amongst people with typical education, motivation, and normal intelligence (Goswami, 2006). On the other hand, a recent study (Callens et al., 2012) has pinpointed that when taking the full cognitive profile of students with DD into account, a quite consistent deficiency on a wide range of tasks, predominantly those involving the speed of processing and retrieval of verbal information from long-term memory, can be identified. Improved reading by training programs in childhood congenital dyslexia (CDD) leads rarely to full restitution, even in children submitted to intensive interventions. A major progress in the treatment of this disorder could originate from the development of complementary approaches that may enhance existing remediation programs by providing rehabilitation benefits that are larger and stable over time.

## Congenital dyslexia and impaired neural activity

Different lines of evidence suggest that early brain development is altered in dyslexic readers. Some imaging studies (Richards et al., 2002) have demonstrated the crucial role of the frontal cortex, for this disorder. For instance, the activity of frontal gyrus was reported to be reduced as shown by functional magnetic resonance imaging (Siok et al., 2008) and near-infrared spectroscopy (Song et al., 2012). Blau et al. (2010) have also shown reduced unisensory responses to letters in the fusiform gyrus and anterior superior temporal gyrus (STG) of dyslexic children. These results are in line with electrophysiological recordings in dyslexic children which have shown that responses to presentation of letter-strings in the occipito-temporal cortex (OTC) were reduced (Maurer et al., 2007).

**Figure 1:**
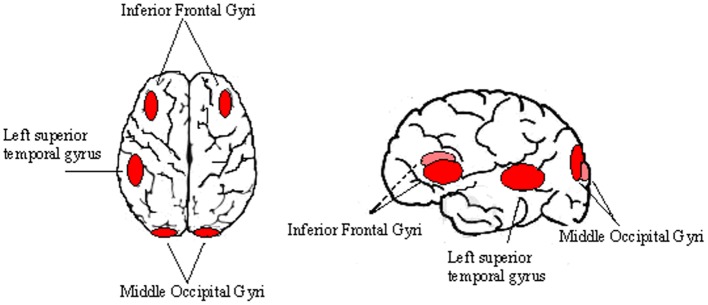
**Cortical areas involved in compensatory processes of childhood dyslexia.** Some cortical regions such as the IFG, the left STG and the middle occipital gyri were reported to be hypo-active in dyslexic readers, while their activity increase with age. This phenomenon seems to be related to compensatory neuroplastic processes.

The identification of left OTC under-activation in young dyslexic readers goes in line with early recruitment of this area in non-impaired readers and the absence of this neural evolution in developmental dyslexia (DD) (Richlan et al., 2011). These brain regions, however, become hyperactive in dyslexic children that are making an effort to overcome their reading dysfunctions (Hoeft et al., 2011). This might reflect compensatory processes in these individuals. In support of this possibility is the finding of Shaywitz et al. (2002) who have found that in dyslexic readers, increasing age was positively correlated with bilateral activation primarily in the inferior frontal gyri (IFG) as well as basal ganglia, left STG and middle occipital gyri as possible physiological correlates of compensation (Figure 1).

A retrospective study has addressed the neural substrates of this compensation more directly by comparing adolescents with dyslexia who were compensated readers versus persistent poor readers (i.e., failed to compensate). For example, Shaywitz et al. (2003) have found an activation of the right superior frontal gyrus during performance of a phonological task that was greater in the compensated as compared to persistently poor readers. Moreover, in a recent longitudinal study over 2.5 years, Hoeft et al. (2011) found that childhood congenital dyslexia (CDD) who at baseline showed greater activation of the right IFG during a rhyme-judgment task showed greater reading improvement over the next 2.5 years. These findings suggest that reading progress in CDD relies on the evolution of neural activity corresponding to frontal and occipito-temporal regions. Hereby, the IFG encompasses the phonological route, having an important role in articulation and naming (Fiez and Petersen, 1998), overt segmentation of speech (Burton et al., 2000) and extrapolation of phonological elements (Gandour et al., 2002). Finally, reduced fractional anisotropy in the left arcuate fasciculus of adults with dyslexia was recently described (Vandermosten et al., 2012). Correlational analyses demonstrated a specific relationship between phoneme awareness and speech perception and integrity of this area. This finding supports the suggestion that this area sustains the dorsal phonological route, and provides a physiological substrate of deranged phonological processing in dyslexia, which is considered to bet he core deficit of this disease.

## Remediation programs and brain plasticity in CDD

Currently, training programs focusing on the deficient aspects of reading skills, such as those involving the retrieval of verbal information from long-term memory and attention (Temple et al., 2003) probably represent the most important instruments for successful treatment of CDD (Gabrieli, 2009). For instance, the recent study of Lovio et al. (2012) shows that 3-h Grapho-Game training, an intervention game developed for the training of letter–sound associations by natural speech (phoneme sounds) and the corresponding letters (Lyytinen et al., 2007), resulted in larger progress in reading-related skills of 6-year-old preschool children as compared to matched controls.

The impact of training on reading skills is explained via a process of neural plasticity involving several brain structures of this children population. Increases in IFG activation following a remedial training have been reported in numerous studies involving dyslexic children. For instance, Temple et al. (2003) showed that after training, activation increases in the left temporo-parietal cortex and left IFG, bringing brain activation in these regions closer to the level seen in normal-reading children. An increased activity following intensive training was observed also in the anterior cingulate gyrus, a brain regions involved in attention (Bush et al., 2000). All these findings show that neural plasticity primed by the adopted remediation program is a key factor in determining the level of reading improvement in dyslexic children.

## tDCS as potential tool for the treatment of CDD

Stimulation with weak direct currents (transcranial direct current stimulation, tDCS) is a non-invasive brain stimulation method, which alters cortical excitability, and activity. Anodal stimulation enhances, whereas cathodal tDCS reduces excitability. The after-effects of stimulation can last for an hour or longer, dependent on stimulation duration, and intensity (Nitsche and Paulus, 2000, 2001; Nitsche et al., 2008). During stimulation, anodal and cathodal tDCS primarily modulate neuronal resting membrane potential. Anodal tDCS results in subthreshold depolarization, while cathodal tDCS hyperpolarizes neurons (Nitsche et al., 2003; Stagg and Nitsche, 2011). Sufficiently long stimulation for some minutes results in after-effects for up to 1 h duration, which resemble alterations of the strength of glutamatergic synapses (Nitsche et al., 2003, 2004). Moreover, a reduction of GABAergic activity might contribute to both, the excitability-enhancing after-effects of anodal, and excitability-diminuishing effects of cathodal tDCS (Stagg et al., 2009). Therefore, tDCS-induce plasticity share some important characteristics with long-term depression (LTD) and long-term potentiation (LTP) induced in animal experiments. LTP represents an important biological substrate of plasticity associated with learning and memory, as well as the reorganization of neuronal circuits after brain injury, being responsible for long-term changes in neuronal circuits (Johnston, 2009).

It is suggested that performance gains induced by behavioral training can be maximized when combined with techniques of cortical neuromodulation, such as tDCS, which induce/increase neuroplasticity (for instance, see Madhavan and Shah, 2012). Accordingly, tDCS has been recently successfully probed for improving rehabilitation of adult patients suffering from stroke symptoms (Schlaug and Renga, 2008), and for improving cognitive functions (Iyer et al., 2005; Kuo and Nitsche, 2012). Of particular relevance for the topic of this paper are studies documenting performance enhancements language learning associated with tDCS. For the language domain, it was reported that anodal stimulation over the posterior part of the left peri-sylvian improved learning of artificial object names (Flöel et al., 2008), and artificial grammar learning was improved by stimulation of the left Broca area in healthy humans (de Vries et al., 2010). On the other hand, performance improvements in linguistic task was also reported in association to cathodal stimulation. For instance, it was recently shown that cathodal stimulation of the primary motor cortex enhances the detection of semantic dissonance (Vicario and Rumiati, 2012). Cathodal tDCS upon the left Posterior Parietal cortex (PPC) seems also able to reduce the variability during the execution of a time reproduction task (Vicario et al., in press). Moreover, there is evidence that cathodal tDCS is able to act as a neuronal noise reducer, thus, facilitating acquisition of executive functions (Antal et al., 2004; Dockery et al., 2009). Therefore, functional improvement accomplished by tDCS might depend not so much only on polarity of stimulation, but could depend on task characteristics, such as learning state, and noisy aspects of information processing, amongst others.

The application of tDCS for the remediation of DD could represent a new frontier of research that can have a significant impact with regard to the current debate on the contribution that neuroscience may provide for education. Given its effect on promoting learning and driving neural plasticity, tDCS could serve as a complementary tool to accompany the standard remediation protocols conceived for CDD, in order to speed up and consolidate neurophysiological changes underlying a behavioral treatment.

With regard to the safety of tDCS in the respective patient population, it should be noticed that tDCS is safe and well tolerated in adults (Nitsche et al., 2008), but so far is rarely applied in children. Therefore, definite information regarding its tolerability in children/adolescents is lacking. At present, the majority of studies using tDCS in children with brain disorders have focused on the treatment of neurologic/psychiatric diseases. Recently the tolerability of tDCS was explored in a pedriatric population suffering from childhood-onset schizophrenia (Mattai et al., 2011). In these patients, 20 min bilateral tDCS to the STG with 2 mA intensity was well tolerated. Thus, safety concerns should not prevent the application of tDCS in children, however, close monitoring for safety aspects should be performed in respective studies.

## Potential tDCS protocols in the treatment of CDD

Taking advantage of current knowledge of functional and structural neural changes caused by intensive reading training programs, it is possible to develop potential treatment approaches that may strengthen compensatory brain reorganization primed by standard remediation programs.

### Intervention on brain areas activated by successful training programs

One possibility is the application of excitability-enhancing anodal tDCS on brain regions which result primarily hypo-active, but become more activated during an intensive training for reading improvement. The rationale of this approach is to support the development of task-related plasticity by tDCS. Brain regions such as the left IFG, whose primary under-activation may reflect a dysfunction in efficient access to lexical and sublexical phonological output representations (Richlan et al., 2009); the primary motor cortex, a region close to the mouth area (Fox et al., 2001), whose activation may reflect compensatory reliance on articulation-based access to phonological word representations (see Richlan et al., 2009 for a complete meta-analysis), but also the bilateral anterior cingulate gyrus (Temple et al., 2003; Keller and Just, 2009), a brain region involved in attention (Bush et al., 2000) and shown to be hypo-active in attention disorders (Bush et al., 1999), are promising targets, because these might be causally linked to the improvement of reading skills.

### Intervention on brain areas typically hypo-active in DD

Beneficial effects might also originate from the application of anodal tDCS upon other regions typically hypo-active in this clinical population, that is the left OTC (Richlan et al., 2011), which is part of the visual reading route (Sandak et al., 2004), and the right hemispheric IFG, whose level of activity constitutes a predictive factor for future reading skills of dyslexic children (Temple et al., 2003). While the left hemispheric intervention can be directly linked to the impact of tDCS on language brain regions, the rationale underlying right hemispheric intervention is that improvements in reading in DD seem to be associated with compensatory mechanisms involving right hemispheric pathways (Turkeltaub et al., 2003).

### Intervention on brain areas involved in visuo-spatial attention mechanisms

A further modality of intervention to be tested could consider the modulation of neural pathways involved in visuo-spatial attention mechanisms. The possible benefit of tDCS over the cingulate gyrus (Temple et al., 2003) and its role on attention (Bush et al., 2000) was already discussed. It has been recently suggested that deficient visual-spatial attention, independent from language-related functions, could contribute to dyslexia (Vidyasagar and Pammer, 2010). Moreover, it has been demonstrated that visuo-spatial training by visual hemisphere-specific stimulation improves reading abilities (Facoetti et al., 2003) in dyslexia. Recently it was reported that anodal stimulation of the right PPC increases training-induced improvement of visual-spatial exploration, as compared to sham tDCS (Bolognini et al., 2010). Thus, according to the suggestion of a visuo-spatial attention alteration in CDD (Vidyasagar and Pammer, 2010), one could expect that the reading skills of these children could improve as result of anodal PPC tDCS.

The application of all these protocols should be done adjunctive to the programmed training interventions, as it was shown that combination of training and tDCS promote a more stable and consistent improvement of cognitive functions (i.e., Madhavan and Shah, 2012).

## Conclusions and precautions

The idea of using tDCS as remediation tool to provide a helpful and efficient instrument to improve the quality of learning in CDD seems to be a promising possibility. However, the development of respective stimulation protocols is hampered by some limitations. First, we actually do not know which brain area is the most promising for this type of intervention. It is also unclear how strong and long the selected brain areas should be stimulated. Furthermore, knowledge about other factors affecting the efficacy of tDCS to improve performance in these patients, such as stimulation period (before/during/after learning) and the type of electrode montage upon the scalp have not been explored so far systematically. To overcome these limitations will be an important endeavor of future studies.

Cohen Kadosh et al. (2012) have recently discussed the ethical issues related to the use of tDCS in children with learning disorders. A central point raised by these authors concerns the limits in assessing safety guidelines for using tDCS in the treatment of higher cognitive functions such as reading or mathematics in children via standard pre-clinical experimental protocols. In fact, the differences in the anatomy and functions of the brain of adults and/or animals might not reveal possible side effects of stimulating a developing brain (Johnson et al., 2010). This brings up the thorny problem of how to develop suitable programs of remediation which may have negligible side effects in children. As argued by Cohen Kadosh et al. (2012), a longitudinal monitoring of cognitive performance and neural functions of dyslexic children treated with this non-invasive brain stimulation technique could provide useful information for assessing the therapeutic effectiveness of the adopted stimulation protocol as well as the presence of side effects.
